# The Benslimane’s Artistic Model for Females’ Gaze Beauty: An Original Assessment Tool

**DOI:** 10.1007/s00266-016-0721-y

**Published:** 2016-12-28

**Authors:** Fahd Benslimane, Laura van Harpen, Simon R. Myers, Fabio Ingallina, Ali M. Ghanem

**Affiliations:** 1 Plastic Surgeon, Private Practice Clinique Benslimane, 7 rue Ahmed Annaciri, Palmiers, 20100 Casablanca, Morocco; 2 Academic Plastic Surgery, Barts and the London School of Medicine and Dentistry, Blizard Institute, Queen Mary University of London, 4 Newark Street, London, E1 2AT UK; 3 Plastic Surgeon, Private Practice Clinica Di Stefano Velona, Via S. Euplio 162, Catania, Italy

**Keywords:** Beauty, Aesthetic, Artistic, Gaze, Eyelid, Eyes

## Abstract

**Background:**

The aim of this paper is to analyze the aesthetic characteristics of the human females’ gaze using anthropometry and to present an artistic model to represent it: “The Frame Concept.” In this model, the eye fissure represents a painting, and the most peripheral shadows around it represent the frame of this painting. The narrower the frame, the more aesthetically pleasing and youthful the gaze appears.

**Materials and Method:**

This study included a literature review of the features that make the gaze appear attractive. Photographs of models with attractive gazes were examined, and old photographs of patients were compared to recent photographs. The frame ratio was defined by anthropometric measurements of modern portraits of twenty consecutive Miss World winners. The concept was then validated for age and attractiveness across centuries by analysis of modern female photographs and works of art acknowledged for portraying beautiful young and older women in classical paintings.

**Results:**

The frame height inversely correlated with attractiveness in modern female portrait photographs. The eye fissure frame ratio of modern idealized female portraits was similar to that of beautiful female portraits idealized by classical artists. In contrast, the eye fissure frames of classical artists’ mothers’ portraits were significantly wider than those of beautiful younger women.

**Conclusion:**

The Frame Concept is a valid artistic tool that provides an understanding of both the aesthetic and aging characteristics of the female periorbital region, enabling the practitioner to plan appropriate aesthetic interventions.

**Level of Evidence III:**

This journal requires that authors assign a level of evidence to each article. For a full description of these Evidence-Based Medicine ratings, please refer to the Table of Contents or the A3 online Instructions to Authors. www.springer.com/00266.

## Introduction

**Fig. 1:**
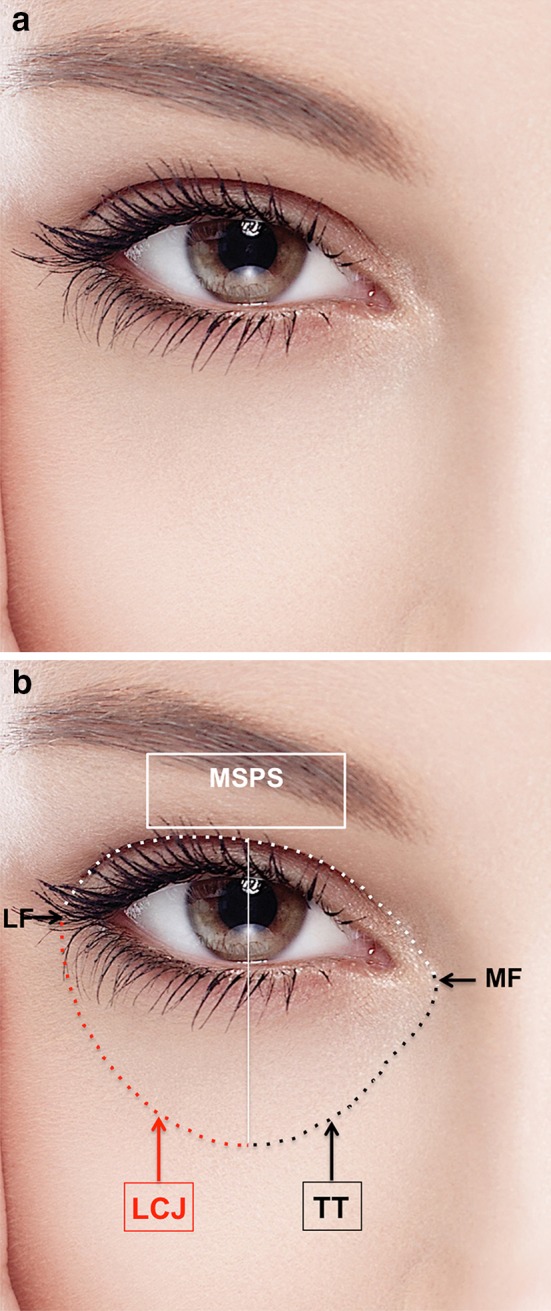
**a** Photograph of the peri-ocular area of a young attractive female model. The eye fissure presents a jaguar-like lateral upward slanting. The sclera presents a snow- white color. The pupil is large in relation to the iris diameter. The upper eyelid crease is in a *low position* resulting in minimal pretarsal skin show. The skin is taut and elastic. The surface area between the eyebrow and the upper eyelid crease is full and convex. The lid–cheek junction is smooth. The transition between the lateral aspect of the nose and the medial aspect of the lower lid is smooth. As a result, the tear trough is barely visible. The lower eyelid appears vertically short. **b** Same model as shown Fig. 1a. The frame of shadows around the eye fissure is highlighted. The superior border of the frame comprises the most superior peripheral shadow (MSPS) between the upper eyelid margin and the eyebrow. It coincides with the upper eyelid crease in this particular model. The inferior border comprises the shadows of the tear trough (TT) medially and the lid–cheek junction (LCJ) laterally in relation to the vertical mid-pupillary line. The lateral convergence of the upper and lower extremities of the frame of shadows defines the lateral frame (LF). The medial convergence of the upper and lower extremities of the frame of shadows defines the medial frame (MF)

It is well established that a youthful attractive face presents with a full upper eyelid [1–14] and a seemingly short lower eyelid in the vertical dimension with a smooth transition at the lid- cheek junction [15–24] (Fig. 1a). However, the aesthetic of the upper and lower eyelids is most often evaluated separately; the aesthetic of the gaze as a whole is rarely analyzed [25–27].

Benslimane developed an artistic model for gaze attractiveness evaluation named the “Frame Concept.” By imagining the eye fissure as a work of art, he views the furthest peripheral shadows surrounding the eye fissure as the frame of this artwork [12] (Fig. 1b). However, there are no objective quantitative measurements to verify this concept, which are necessary to enable a reliable, reproducible clinical application.

The aim of this study is to validate scientifically the Frame Concept using anthropometric ratio analysis [28, 29]. The anthropometric ratio was measured on modern icons of female beauty, historical references of beautiful youthful female faces, and on historical references of older female faces.

## Materials and Methods

The study included a comprehensive literature review of the features that make the gaze appear attractive. The senior author arrived at the Frame Concept after analyzing 454 photos of various faces published in a photographic art book [30] and 1000 portraits from modern photography magazines and the Internet. He also compared photographs from the distant past of 339 patients to their recent headshots, which were taken by the senior author. The photographs were incorporated into PowerPoint to facilitate analytic comparison. (PowerPoint, Windows, California).

The frame’s borders are composed of the following lines: The superior border comprises the most superior peripheral shadow between the upper eyelid margin and the eyebrow. The inferior border comprises shadows of the tear trough medially and the lid–cheek junction laterally in relation to the vertical mid-pupillary line. The lateral convergence of the upper and lower extremities of the frame defines its lateral aspect. The medial convergence of the upper and lower extremities of the frame defines its medial aspect (Fig. 1b).

The senior author terms this concept as the “Frame Concept.” He observed and hypothesized that the narrower the frame, the more youthful and attractive the gaze.

To validate this concept in terms of modern and historical perception of beauty, classical paintings and modern photographs of “idealized” female images of beauty in these respective eras were studied.

**Fig. 2:**
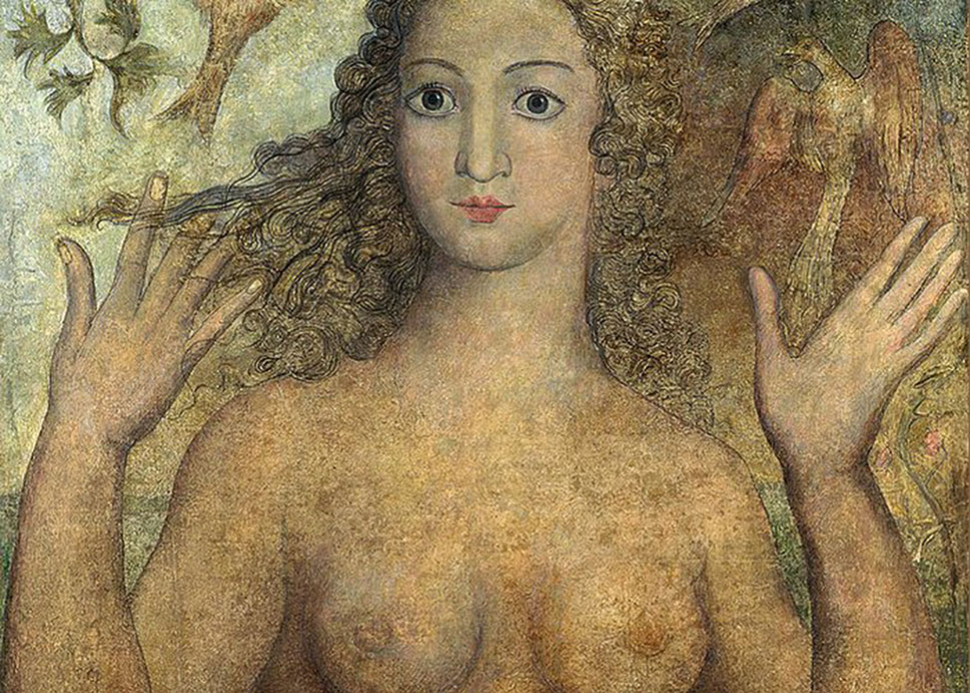
“Eve Naming the Bird” completed in 1810 by William Blake. She presented the lowest frame ratio (1.4) in Group II

**Fig. 3:**
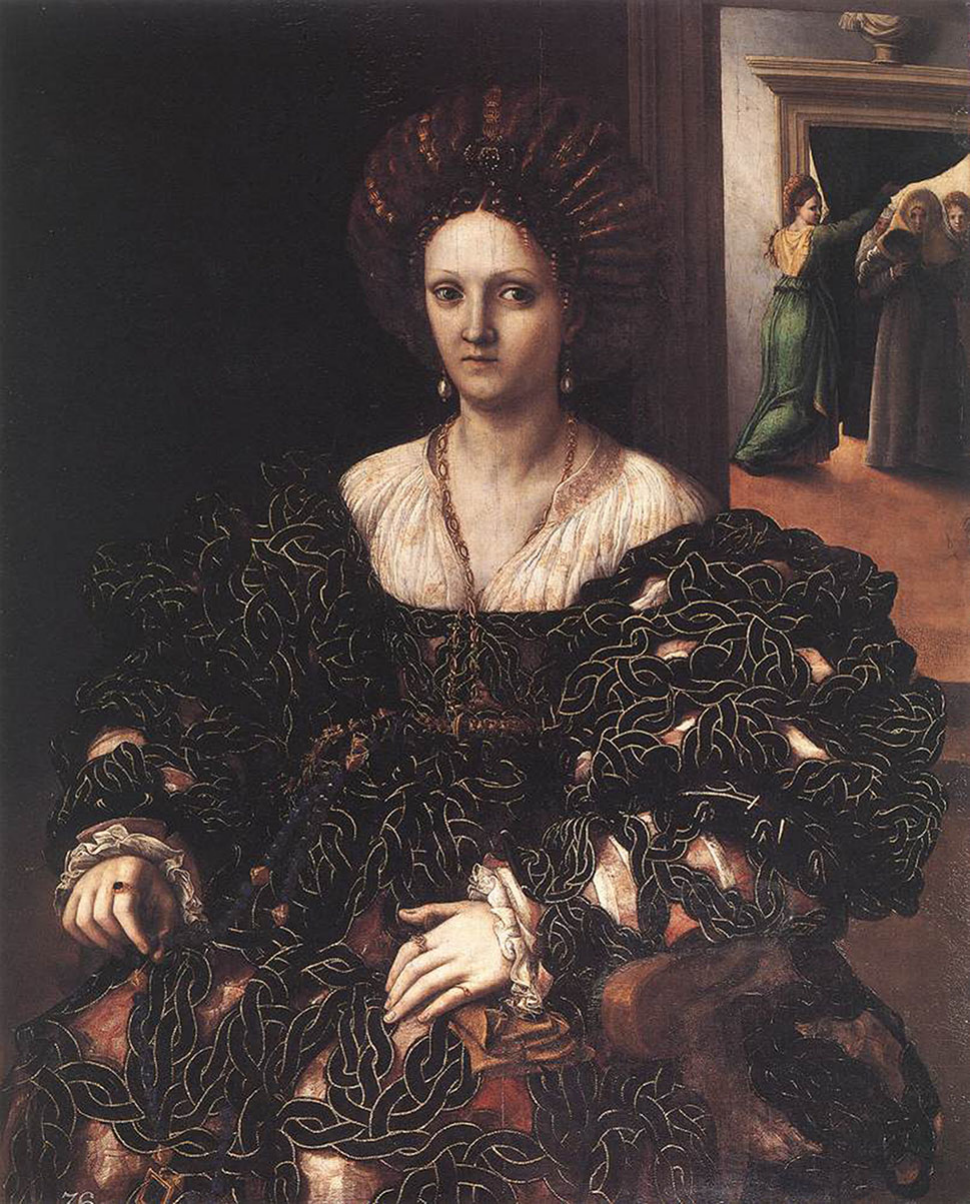
“Portrait of Isabella d’Este” completed in 1536 by Giulio Romano. She presented the highest frame ratio (2.5) in Group II

**Fig. 4:**
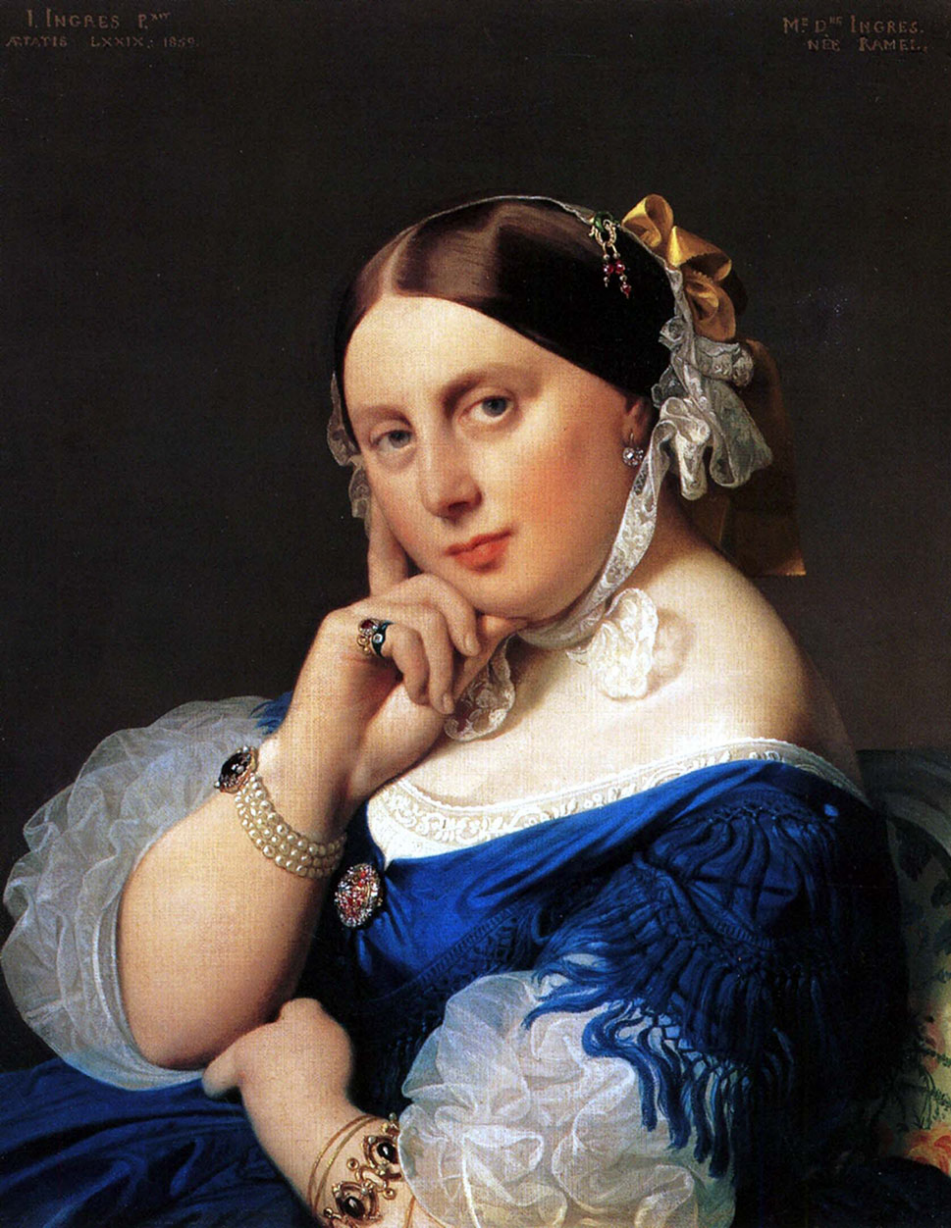
“Madame Ingres” completed in 1877 by Jean Auguste Dominique Ingres. She presented the lowest frame ratio (1.6) in group III

**Fig. 5:**
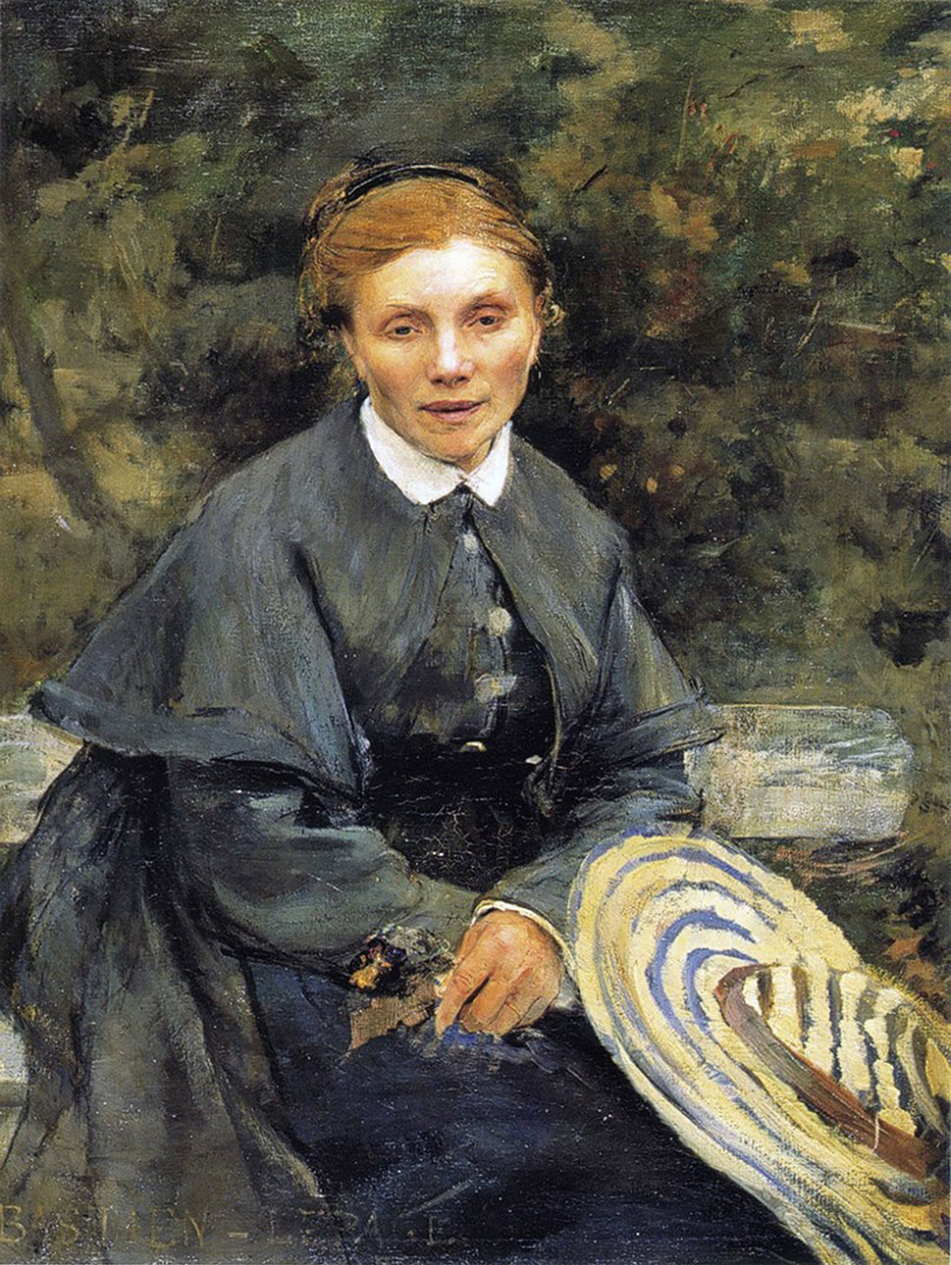
“Portrait of my mother” completed in 1877 by Jules Bastien-Lepage. She presented the highest frame ratio (3.8) in Group III

Three groups were selected as follows: Miss World 1993–2012 (Group I—Table 1), classical art beautiful women (Group II—Table 2; Figs. 2, 3), and classical artists’ mothers (Group III—Table 3; Figs. 4,5). The inclusion criterion was that the images were anteroposterior or oblique views of less than 15-degrees after Park and Hwang [29]. A total of 41 images were collected including 20 from Group I, 10 from Group II, and 11 from Group III.

**Table 1:** Frame ratio measurement on Miss World from 1993 to 2012 (Group I)

Miss World year	Right eye frame ratio	Left eye frame ratio
1993	1.91	2.0
1994	1.97	2.0
1995	1.86	2.2
1996	2.37	2.7
1997	1.92	1.8
1998	2.18	1.9
1999	2.20	2.1
2000	1.88	2.3
2001	2.13	2.3
2002	2.47	2.4
2003	2.03	1.8
2004	2.06	2.1
2005	1.80	1.9
2006	2.67	2.5
2007	2.06	2.0
2008	2.60	2.8
2009	2.58	2.4
2010	2.09	2.3
2011	2.21	2.6
2012	1.85	2.4

**Table 2:** Frame ratio measurement on beautiful young women painted from the 16th to the 19th century (Group II)

Painting name	Artist	Year	Right eye frame ratio	Left eye frame ratio
Portrait of Isabella d’Este	Giulio Romano	1536	2.5	2.4
La bella	Vecellio Tiziano	1536	2.3	2.4
Portrait de femme	Paolo Veronese	16th Century	2.4	2.4
A young woman and her little boy	Agnolo Bronzino	1572	1.7	1.6
Eleonor of Teledo with her son Giovanni de Medici	Agnolo Bronzino	1545	1.7	1.7
Lucrezia Panciatichi	Agnolo Bronzino	1572	1.7	1.6
The Birth of Venus	Sandro Botticelli	1872	1.9	2.1
Eve naming the bird	William Blake	1810	1.4	1.2
Lady with a Fur	El Greco	1580	2.0	2.3
Mona Lisa	Leonardo da Vinci	1503	2.3	2.5

**Table 3:** Frame ratio measured on Artists’ Mother painted from the 17th to the 20th Century (Group III)

Painting name	Artist	Year	Right eye frame ratio	Left eye frame ratio
Portrait of my parents	Felix-Vallotton	1886	2.7	2.3
Portrait of my mother	Guido Reni	1620	2.6	3.0
Anne Constable	John Constable	1804	2.2	2.1
Madame Ingres	Jean Auguste Dominique Ingres	1814	1.6	2.1
Portrait of my mother	Henryk Rodakowski	1853	3.0	2.9
Madame Manet	Edouard Manet	1866	2.4	2.4
Portrait of my mother	Jules Bastien-Lepage	1877	3.8	3.1
Anna Cornelia van Gogh	Vincent van Gogh	1888	2.3	2.4
Painter and his mother	Arshile Gorky	1936	2.1	2.5
Portrait of my mother	Erik Wilson	1937	2.3	2.3
Portrait of my mother, 18 and 88 age old	Tom Phillips	1989	3.1	3.5

**Fig. 6:**
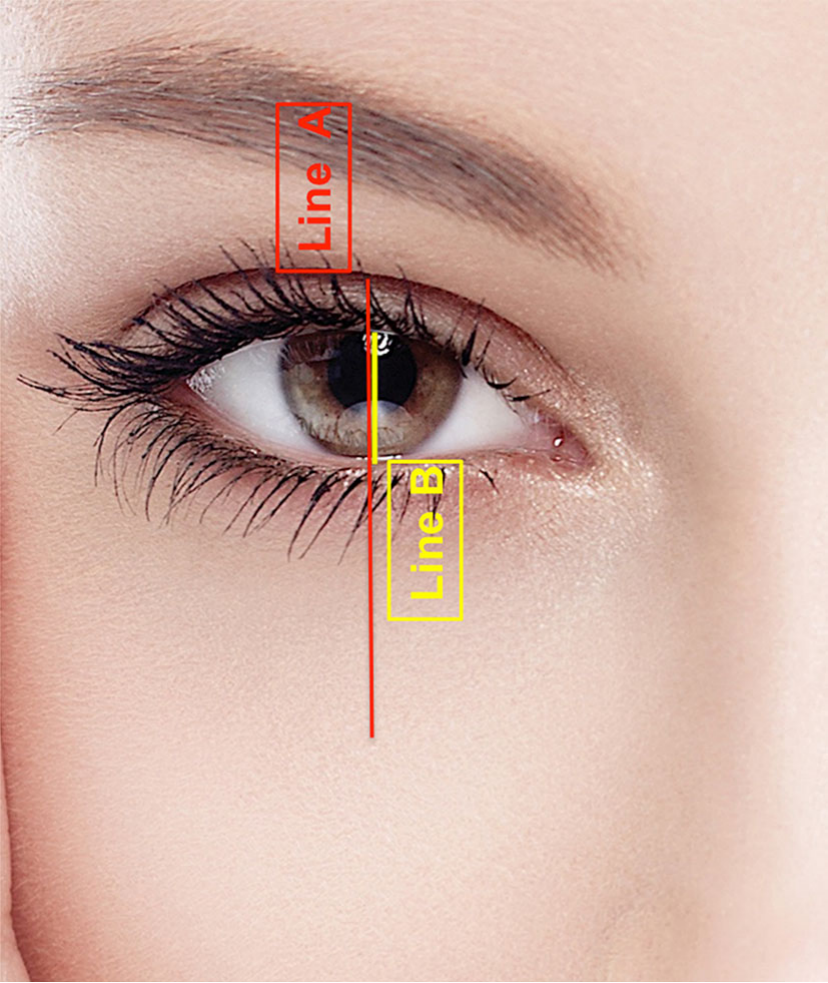
The frame anthropometric measurements: Distance A (*red line*): The vertical height of the frame of shadows defined as the vertical distance measured at the mid-pupillary line between the inferior border of the frame (lid–cheek junction) and the superior border of the frame (The most superior peripheral shadow of the upper eyelid between the eyelid margin and the eyebrow).Distance B (*yellow line*) is defined as the vertical height of the eye fissure measured at the mid-pupillary line. Frame ratio is defined as A/B

**Fig. 7:**
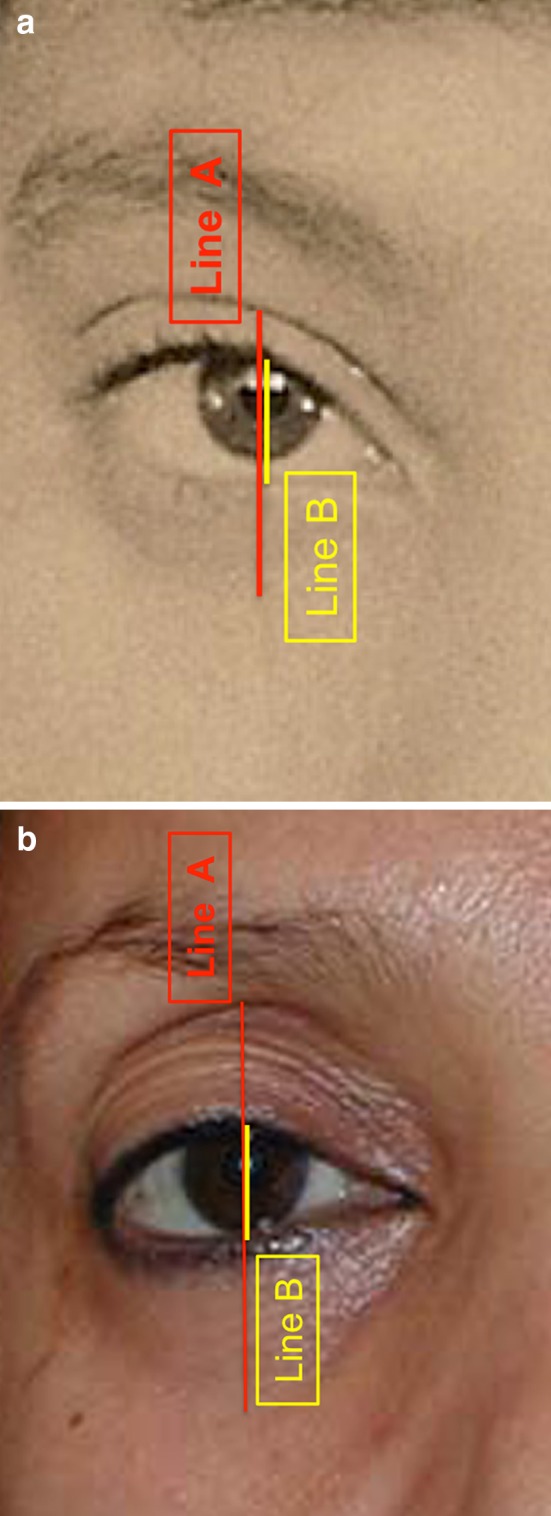
**a** Youthful example is evident in this patient’s photograph when she was 16. The upper eyelid crease is in a low position. Its shadow defines the upper half of the frame. The inferior border of the frame coincides with the highly positioned lid–cheek junction. This configuration results in a short distance A (*red line*) and a small ratio A/B. **b** Photograph of the same patient at the age of 62. Her upper eyelid presents an increased pretarsal/preseptal skin show, which results in an increase of shadows in the upper eyelid area. The lid–cheek junction is in a relatively low position. The long upper and lower eyelids result in a long distance A and a high ratio A/B

**Fig. 8:**
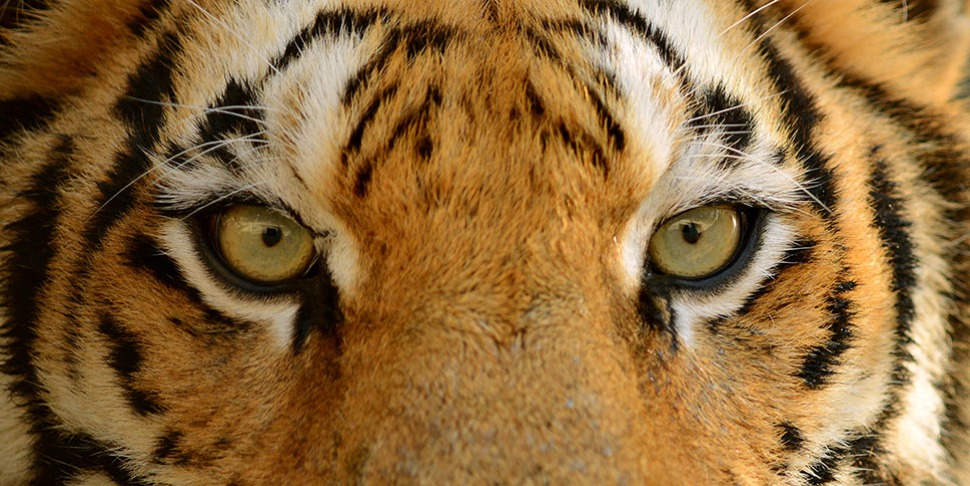
The fascination of Mankind with the gaze of felines has been attributed to the particular gray-green–blue color of their iris and the lateral upward slanting of their eye fissure. However, the absence of shadows around the eyes of felines might also contribute to our fascination with their gaze. Felines’ eyes seem to be directly planted onto their forehead. This gives an intense light concentration in their eye fissure with a unique hypnotizing effect

The frame anthropometric measurements were calculated according to the following distances: Distance A: The mid-pupillary vertical distance between the inferior border of the frame (lid–cheek junction) and the superior border of the frame (most superior peripheral shadow of the upper eyelid between the eyelid margin and the eyebrow). Distance B: The vertical height of the eye fissure measured at the mid-pupillary line. Frame ratio was defined as A/B (Figs. 6, 7, 8). All images were analyzed using ImageJ version 1.46r.

**Fig. 9:**
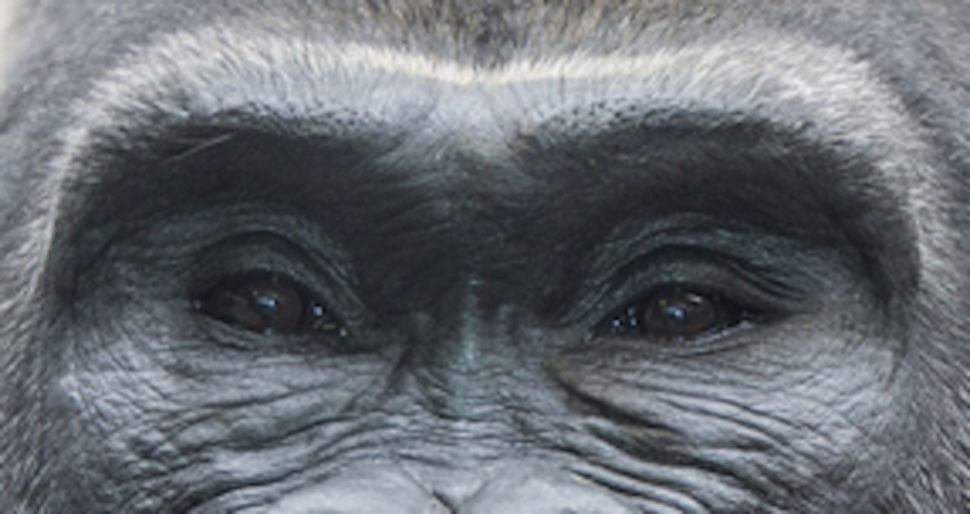
Hollowness around the eyes of primates hampers the light reflection in their eye fissure. The shadows around the eye fissures of primates may contribute to the discomfort some may feel when gazing into their eyes. The similarity between primates and ourselves as we age might disconcert us

The senior author hypothesized that this anthropometric ratio would correlate with female gaze beauty and apparent youthfulness. The smaller the ratio, the more youthful and attractive the gaze will appear. Group I was analyzed to identify the ideal frame ratio based on modern acknowledged models of beauty. Groups I and II were compared to test any change in the ratio with observer perceptions of beauty over the centuries. Groups II and III were compared to understand and evaluate the change of the frame characteristics during the female aging process. Statistical analysis was performed by independent samples *t* test using IBM SPSS^®^ Ed 21 software.

## Results

### Frame Characteristics in Modern Acknowledged Models of Female Beauty

By analyzing the frame ratio of 20 consecutive Miss World photographs (Group I), the average frame ratio was found to be (Mean SD) 2.1 ± 0.3 for the right eye and 2.2 ± 0.3 for the left eye. The lowest frame ratio (1.8) was measured in Miss World 2005. The highest frame ratio (2.7) was measured in Miss World 2006.

### Frame Ratio and Perception of Beauty Across the Centuries

By analyzing the frame ratio of ten portraits of classical artists’ paintings of beautiful women (Group II), the average frame ratio was found to be (Mean SD) 2.0 ± 0.4 for the right eye and 2.0 ± 0.5 for the left eye. The lowest frame ratio (1.4) was measured in “Eve Naming the Bird” by William Blake completed in 1810 (Fig. 2). The highest frame ratio (2.5) was measured in “Portrait of Isabella d’Este” by Giulio Romano completed in 1536 (Fig. 3). Using an independent samples *t* test, there was no statistically significant difference in the mean of frame ratio of either eyes between modern idealized females and those idealized in the classical portrait paintings—*t*(28) = 1.29, p = 0.2 and *t*(28) = 1.497, p = 0.146 for the right and left eye frame ratio, respectively.

### Frame Ratio and the Female Aging Process

The average frame ratio of the eleven portrait paintings of classical artists’ mothers (Group III) was 2.6 ± 0.5 for the right eye and 2.6 ± 0.6 for the left eye. The lowest frame ratio (1.6) was measured in “Madame Ingres” by Jean Auguste Dominique Ingres completed in 1814 (Fig. 4). The highest frame ratio (3.8) was measured in ‘Portrait of my mother’ by Jules Bastien-Lepage completed in 1877 (Fig. 5).

Using an independent samples *t* test, there was a statistically significant difference in the mean frame ratio for both eyes between classical paintings depicting beautiful young females (Group II) and those depicting older graceful females such as the artists’ mothers (Group III)—*t*(19) = −2.599, p = 0.018 and *t*(19) = −2.909, p = 0.146 for the right and left eye frame ratio, respectively.

## Discussion

The role of the human gaze in every circumstance from social to emotional is undeniable. It is therefore important to identify the characteristics that make it attractive or not. Martin et al. showed that the blue-eyed beauty stereotype does exist. However, other colors were rated as attractive as blue eyes [25]. The “snow-white” scleral color and large-appearing pupil were also identified as significantly correlating with youthfulness [25]. The Jaguar-like upward slanting of the lower lid was also reported to influence gaze beauty [26, 27].

Changing scleral color and modifying the pupil’s width to achieve a more attractive and youthful gaze may not be part of the tools available to aesthetic surgeons. Changing the shape of the eye fissure is possible with surgical adjustment of the lower eyelid or lateral canthal angle [31]. In this article, the authors present and validate an original artistic model, the Frame Concept, which describes a new specific and measurable quality of gaze attractiveness. This concept may facilitate selecting appropriate patients and planning “frame narrowing” for periorbital reconstruction and beauty enhancement.

This model considers the eye fissure as a painting: a work of art. At the same time, the most peripheral shadows around the eye fissure is considered as the “frame” of the painting. The narrower the frame, the more attractive and youthful the gaze appears.

Analyzing the Frame Concept in portraits of 20 consecutive Miss World winners identified a universal ideal frame ratio as these twenty females represented all major human ethnicities (7 Europeans, 6 Asians, 3 South Americans, 2 North Americans, and 2 Afro-Caribbeans). The ideal frame ratio in this group was 2.15 with a range of 1.8 to 2.7. There could be ethnic-specific ideals that can be easily identified with standardized anthropometric measurements based on the methodology of this work. This is, however, beyond the scope and purpose of validating the frame concept in this article. Equally, analyzing the frame in 10 of the world’s classical historical paintings would identify any change of perceived beauty of the human female gaze across times (Paintings were completed between the 16th and the 19th Centuries).

Understanding the universal concepts of beauty across racial and time variation is of a great importance for modern aesthetic surgeons. Rhee and Woo demonstrated that perception of the eyes’ beauty is race dependent. In contrast with many Asians, Caucasians do not “seem to prefer big, round eyes.” Instead, they seem to prefer a “Western type” eye shape with a jaguar-like upward slanting position of the lower eyelid laterally [27].

Loeb (15), Flowers (16), Hamra (16), and others (17-24) pointed out the importance of a smooth lid–cheek junction.

Mendelson described a youthful lower eyelid as vertically short in appearance [19]. Its lengthening widens the frame in its lower portion which creates a shadow.

**Fig. 10:**
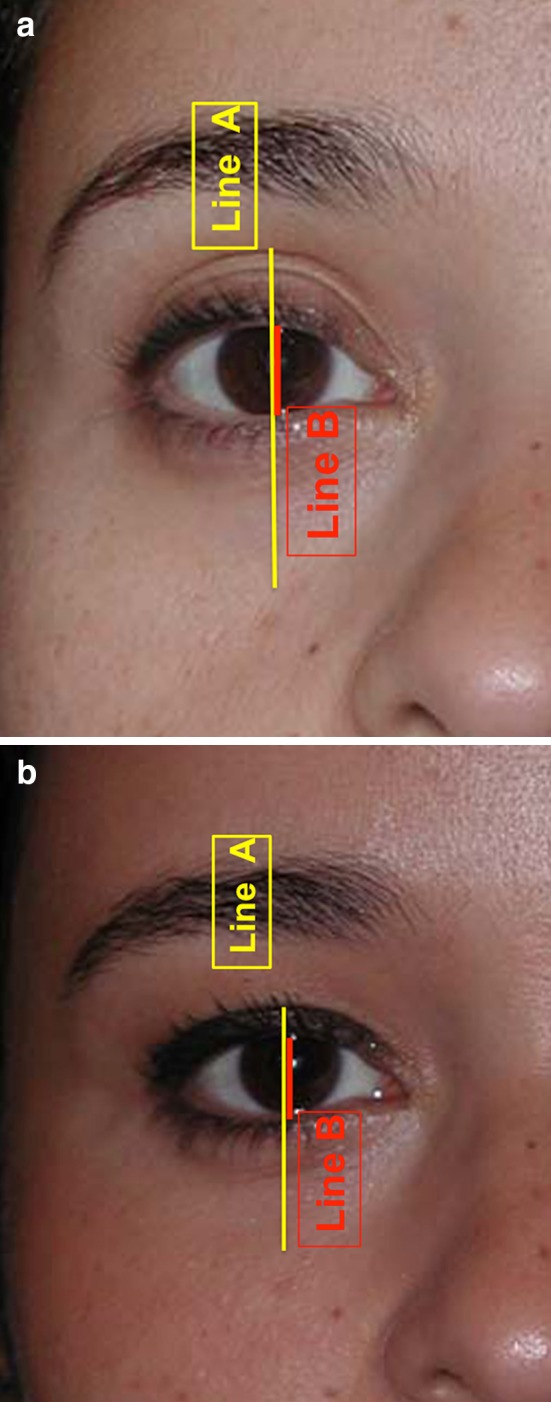
**a** Photograph showing the pre-frame narrowing on the right eye of a patient. The most superior peripheral shadow does not coincide with the upper eyelid crease but with a higher extra fold, which corresponds to the posterior invagination of the upper eyelid’s skin. The lower eyelid appears long. **b** Photograph showing the post-frame narrowing on the right eye of the same patient. Upper eyelid micro fat grafting erased the extra upper eyelid fold and lowered the upper eyelid crease. The lower eyelid appears shorter. Distance A has been lowered resulting in a lower frame ratio

**Fig. 11:**
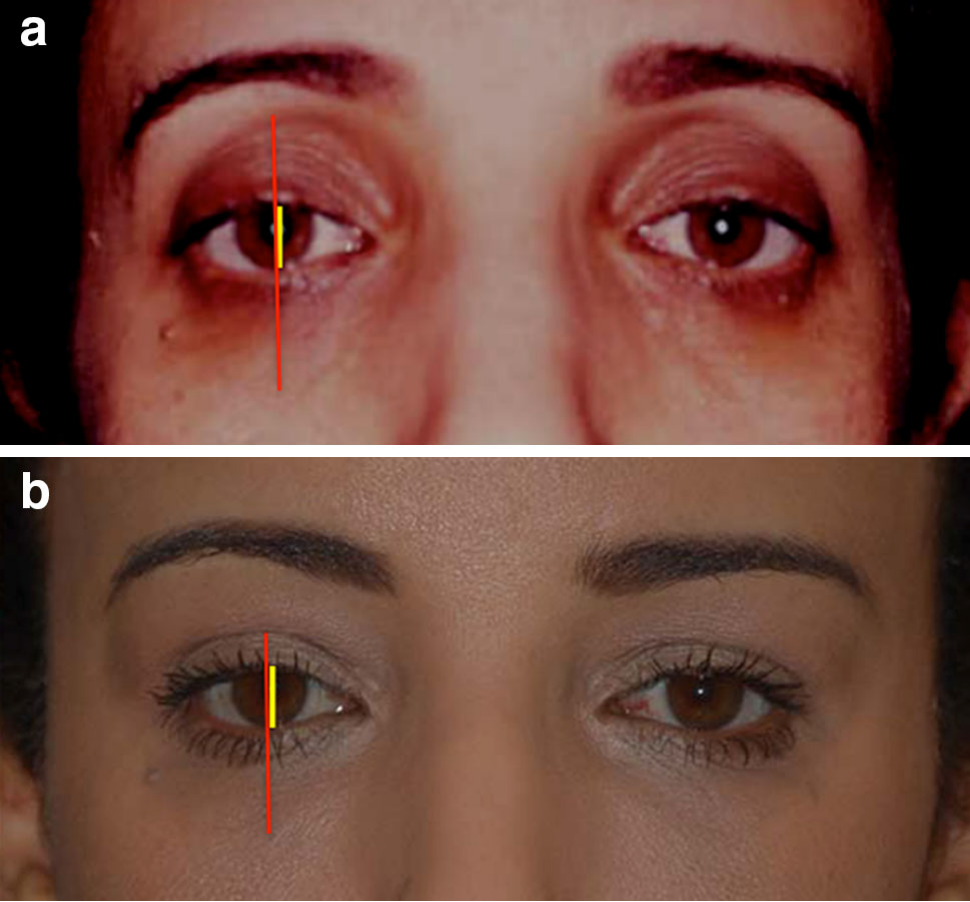
**a** Photograph showing the pre-frame narrowing on a patient. The pretarsal/preseptal skin show is large extending from the upper eyelid margin to the under surface of the upper orbital rim. The lower eyelid appears long. The shadows around the eye fissure create a distracting effect from focusing on the eyes. **b** Photograph showing the post-frame narrowing on the same patient. Upper eyelid micro fat grafting has filled the hollowness between the upper border of the tarsus and the orbital rim. The upper eyelid crease has been lowered. The upper eyelids’ aspect has changed from concave to convex, which has resulted in the elimination of the shadows around the eye fissure and a better focus on the eyes

Little [1], Berman [2] Hwang et al. [3], Fagien [4], Trepsat [5], Rohrich et al. [6], among others [7–14] described a youthful attractive upper lid as full and convex. Coleman demonstrated that addressing the additive component of aging (skin excess), ignoring the substractive component (fat atrophy and volume loss), may lead to a less youthful and attractive appearance [7]. Lambros pointed out the limitations of traditional blepharoplasties and brow lifts, and the changes in the relationships between subcutaneous volume and skin quality—[9, 11]. Knoll et al. demonstrated that an increase in pretarsal show leads to an increased perception of tiredness and sadness [32]. This corroborates with our findings: The increased pretarsal show results in an increased shadow on the upper eyelid and widens the frame in its upper portion. The perception of increased tiredness may be the result of an increased shadow, which might distract the attention of the perceiver from focusing on the eye fissure (Fig. 7a, b). The absence of shadows around the eyes of felines might contribute to our fascination with their gaze as pointed out by Benslimane in his work with animal models. Conversely, the shadows around the eye fissures of primates may contribute to the discomfort some may feel when we gaze into their eyes. The similarity between primates and ourselves as we age might disconcert us [12] (Figs. 8, 9). Guyuron demonstrated the strong association between senescence-related enophthalmos and eyelid ptosis [33] (Fig. 7b). This is in line with our finding: The upper eyelid crease becomes higher resulting in an elongated and hollowed upper eyelid area as ptosis secondary to levator dehiscence evolves [31]. This results in the augmentation of the ratio A/B. Consequently, the first surgical goal to achieve an attractive gaze might be to think in terms of frame narrowing by ptosis repair when needed and volume enhancement of the upper and lower eyelid with or without limited skin resection (Figs. 10, 11). Hence, the frame concept can be used to both facilitate the doctor–patient subjective artistic discussions of aesthetic goals and the more objective and overlapping rejuvenation procedure planning.

## Conclusion

The Frame Concept is an aesthetic artistic model to evaluate the beauty of the female gaze. It is a user-friendly tool to help direct the patients’ attention to the shadow areas around her eye fissures. This enhances their capacity to broaden their perspective, perceiving the eye within an artistic framework.

Incorporating the frame ratio model helps restore a youthful appearance by narrowing the frame of shadows, thus allowing for an improved focus on the work of art within it: the eye. Future Frame Concept studies with a wider cohort are necessary to establish normative data.

The future studies should consider the variations among races in order to tailor frame narrowing in accordance with the patient’s ethnicity.
